# Long splenic flexure carcinoma requiring laparoscopic extended left hemicolectomy with CME and transverse-rectal anastomosis: technique for a modified partial Deloyers in 5 steps to achieve enough reach and preserving middle colic vessels

**DOI:** 10.1007/s00423-021-02240-7

**Published:** 2021-07-16

**Authors:** Salomone Di Saverio, Kostantinos Stasinos, Weronyka Stupalkowska, Umberto Bracale, Pierpaolo Sileri, Antonio Giuliani, Giuseppe Nigri, Efstratios Kouroumpas, James M. D. Wheeler, Giovanni Domenico Tebala, Francesco Di Marzo, Belinda De Simone, Carlos Pastor Idoate, Nicola De Angelis, Roberto Cirocchi, Patricia Tejedor

**Affiliations:** 1 Department of General Surgery, University Hospital of Varese, University of Insubria, Varese, Italy; 2 Cambridge Colorectal Unit, Cambridge University Hospitals NHS Foundation Trust, Addenbrooke’s Hospital, Cambridge Biomedical Campus, Hills Road, Box 201, Cambridge, CB2 0QQ UK; 3 University of Naples, Naples, Italy; 4 University Vita Salute San Raffaele, Milan, Italy; 5 San Carlo Hospital, Potenza, Italy; 6 La Sapienza University of Rome, Rome, Italy; 7 Colorectal Unit, Addenbrookes Hospital, Cambridge, UK; 8 Oxford University Hospital, Oxford, UK; 9 Estar Toscan, San Sepolcro Hospital, Florence, Italy; 10 University of Paris, Paris, France; 11 Colorectal Unit, Clinica Universidad de Navarra, Madrid, Spain; 12 Henri Mondor University of Creteil, Paris, France; 13 University of Perugia, Perugia, Italy; 14 University Hospital ‘Gregorio Marañón’, Madrid, Spain

**Keywords:** Left extended colectomy, Complete mesocolic excision, Deloyers procedure, Splenic flexure carcinoma, Embryology, Colonic derotation

## Abstract

**Introduction:**

This How-I-Do-It article presents a modified Deloyers procedure by mean of the case of a 67-year-old female with adenocarcinoma extending for a long segment and involving the splenic flexure and proximal descending colon who underwent a laparoscopic left extended hemicolectomy (LELC) with derotation of the right colon and primary colorectal anastomosis.

**Background:**

While laparoscopic extended right colectomy is a well-established procedure, LELC is rarely used (mainly for distal transverse or proximal descending colon carcinomas extending to the area of the splenic flexure). LELC presents several technical challenges which are demonstrated in this How-I-Do-It article.

**Technique and methods:**

Firstly, the steps needed to mobilize the left colon and procure a safe approach to the splenic flexure are described, especially when a tumor is closely related to it. This is achieved by mobilization and resection of the descending colon, while maintaining a complete mesocolic excision to the level of the duodenojejunal ligament for the inferior mesenteric vein and flush to the aorta for the inferior mesenteric artery. Subsequently, we depict the adjuvant steps required to enable a primary anastomosis by trying to mobilize the transverse colon and release as much of the mesocolic attachments at the splenic flexure area. Finally, we present the rare instance when a laparoscopic derotation of the ascending colon is required to provide a tension-free anastomosis. The resection is completed by delivery of the fully derotated ascending colon and hepatic flexure through a suprapubic mini-Pfannenstiel incision. The primary colorectal anastomosis is subsequently fashioned in a tension-free way and provides for a quick postoperative recovery of the patient.

**Results:**

This modified Deloyers procedure preserves the middle colic since the fully mobilized mesocolon allows for a tension-free anastomosis while maintaining better blood supply to the mobilized stump. Also, by eliminating the need for a mesenteric window and the transposition of the caecum, we allow the small bowel to rest over the anastomosis and the mobilized transverse colon and reduce the possibility of an internal herniation of the small bowel into the mesentery.

**Conclusions:**

Laparoscopic derotation of the right colon and a partial, modified Deloyers procedure preserving the middle colic vessels are feasible techniques in experienced hands to provide primary anastomosis after LELC with improved functional outcome. Nevertheless, it is important to consider anatomical aspects of the left hemicolectomy along with oncological considerations, to provide both a safe oncological resection along with good postoperative bowel function.

**Supplementary Information:**

The online version contains supplementary material available at 10.1007/s00423-021-02240-7.

## Introduction

Splenic flexure (SF) carcinoma represents a technical challenge for the colorectal surgeons both for the difficulty of the anatomical location and dissection from the surrounding organs (spleen, tail of the pancreas, duodenojejunal flexure), but also and above all for the reconstruction and the vascular anatomy of the remaining colon if an extended left hemicolectomy is performed (usually proximal/mid transverse colon remains after resection to be anastomosed with the upper rectum). Surgical options for a SF carcinoma can either be an extended right hemi (and reconstruction by ileo-descending anastomosis), a segmental resection of the SF, or an extended left hemicolectomy which requires a reconstruction with a full Deloyers procedure.

While the extended right is a preferable choice for cancers located in the distal third of the transverse colon and/or just above the SF, an extended left hemicolectomy with complete mesocolic excision (CME) along the inferior mesenteric vein (IMV) and inferior mesenteric artery (IMA) seems to be a better option for tumors located in the proximal descending colon and/or just below the SF. The option of performing a segmental resection is usually reserved for elderly patients with tumors located across or just below the SF, due to the thought that the resection will be limited and may not be the best oncological technique (Table 1). However, there is a lack of strong evidence in favor of just one of the mentioned approaches for the splenic flexure carcinoma.

**Table 1:** Practical algorithm on SF carcinoma intraoperative decision-making

Patients’ characteristics	Type and extent of surgical resection	Type of reconstruction
Elderly patient (> 70 yo), (with severe comorbidities, high ASA score) with locally advanced carcinomas and cN + , located proximal, distal or across the SF	Segmental SF resection	Colo-colic anastomosis
Patients < 70 yo, patients with locally advanced carcinomas and cN + , located in the distal third of the TC ± across the SF	Extended right colectomy with CME and CVL of the middle colic vessels	Ileo-descending or ileo-sigmoid anastomosis
Patients < 70 yo, patients with locally advanced carcinomas and cN + , located across the SF ± proximal descending colon	Extended left colectomy with CME and high ligation of the IMV and IMA	Colorectal anastomosis (between HF or proximal TC and the upper rectum) (possibility of performing a standard or modified Deloyers procedure)
Patients < 70 yo, with early colon carcinomas and cN-, located in the distal third of the TC ± across the SF	Segmental SF resection (ICG guided if ICG available) radical lymph node dissection along the middle colic and left colic vessels	Colo-colic anastomosis

*TC*, transverse colon; *SF*, splenic flexure; *ICG*, indocyanine green; *HF*, hepatic flexure; *yo*, years old; *IMV*, inferior mesenteric vein; *IMA*, inferior mesenteric artery; *CVL*, central vascular ligation

Vascular supply and the presence of the middle colic vessels, often represent a technical obstacle to a tension-free and well-vascularized colorectal anastomosis. Deloyers procedure has been described to overcome these anatomical issues, including the sacrifice of the middle colic vessels and involved rotation about the ileocolic pedicle (placing the cecum in the right upper quadrant) or creating a defect in the mesentery and delivering the right colon through to meet the rectum. In selected cases with favorable anatomy and with wider mobilization of the transverse mesocolon, fully up the origin of the middle colic vessels from the superior mesenteric axis, below the body of the pancreas, a modification of the original Deloyers procedure maybe effectively performed with preservation of the middle colic vessels, therefore achieving a tension-free and even better vascularized anastomosis between the proximal transverse colon and the upper rectum.

## Case presentation

**Fig. 1:**
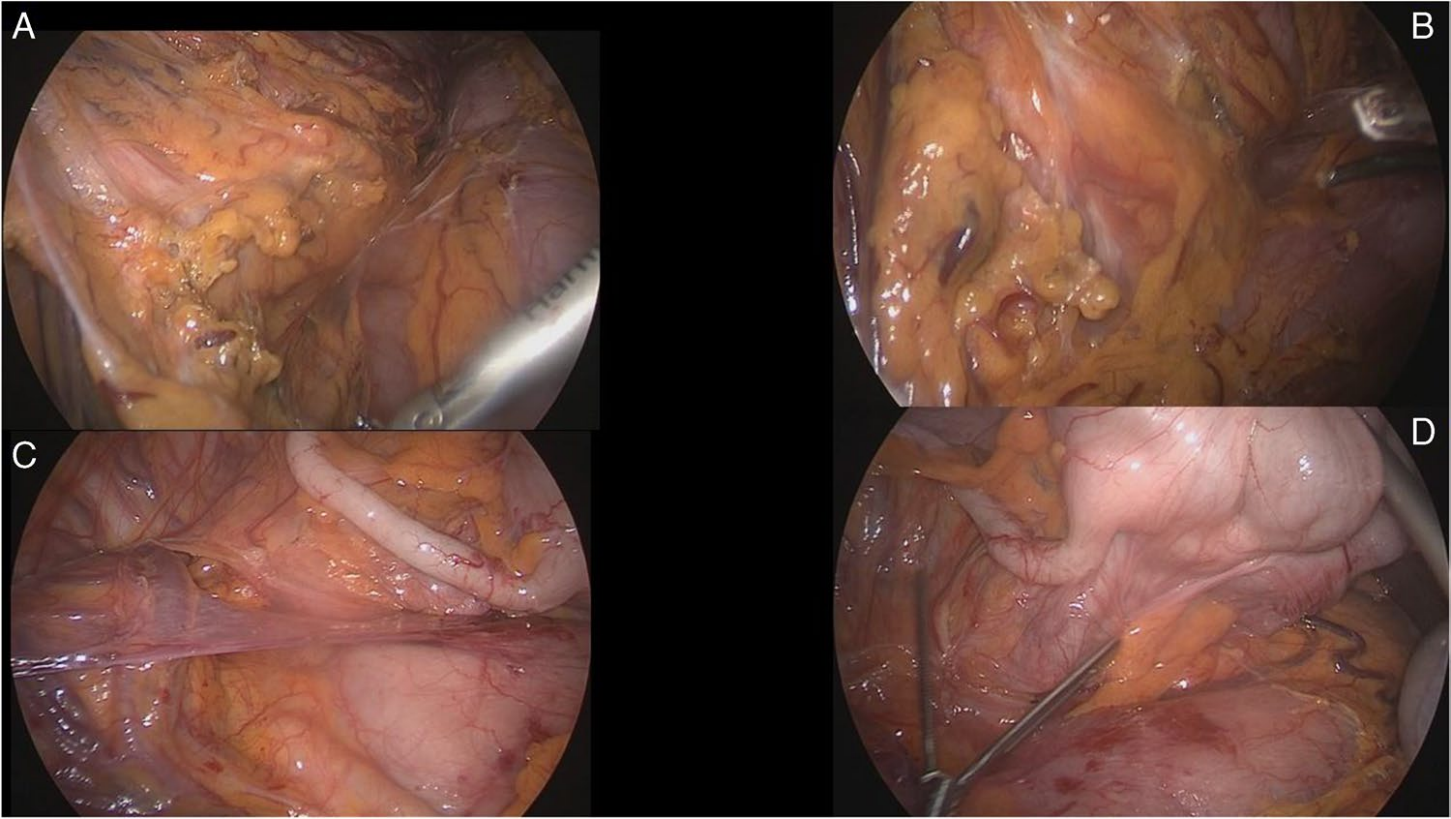
Panel **A**—Entered lesser sac and dividing the embryologic adhesions of the TC mesentery. Panel **B**—Divide and open the Fredet’s fascia. Panels **C** and **D**—Toldt’s fascia is fully mobilized and the right colon is going up to reach the RUQ (visible the appendix over the duodeno-pancreatic head

**Fig. 2:**
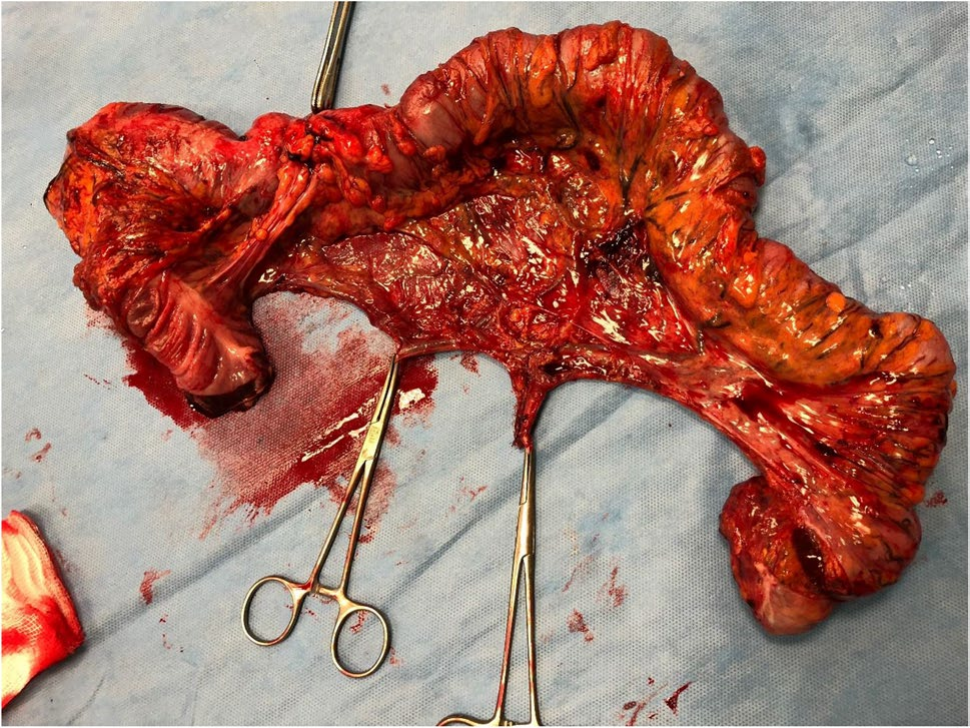
Specimen with IMV and IMA stump taken at their origin

**Fig. 3:**
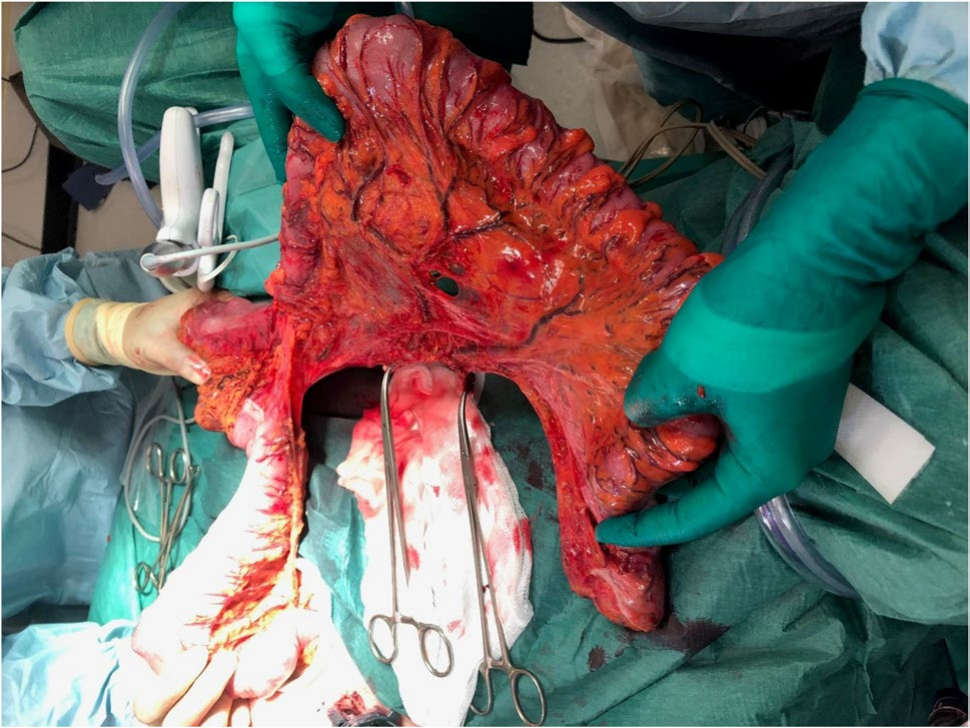
Specimen outside its length is demonstrated and transverse colon is exteriorized up the hepatic flexure, which is visible at the level of the wound protector (suprapubic incision)

**Fig. 4:**
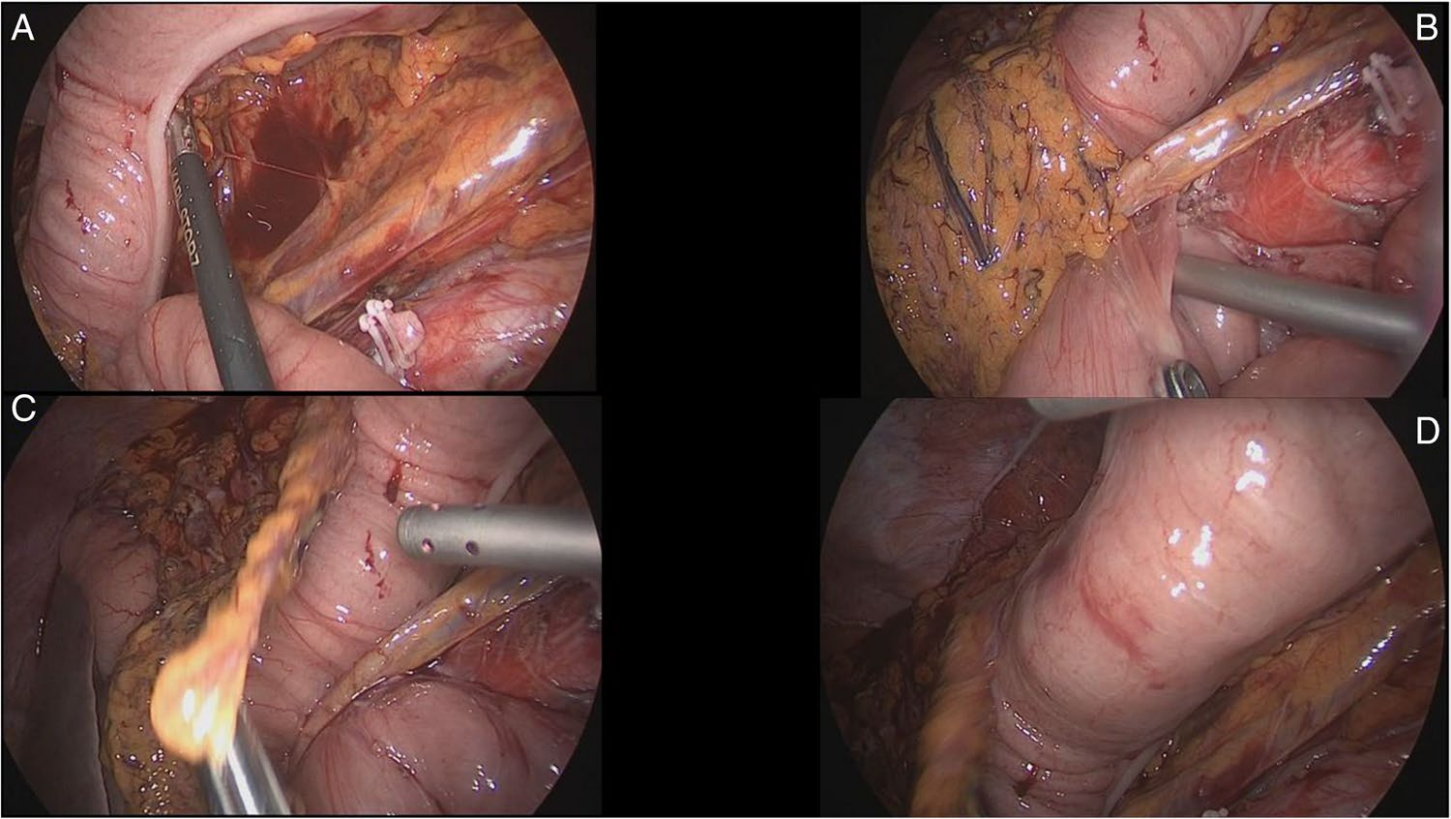
Panel **A**—Colorectal anastomosis already stapled, SB on the right, no internal hernia, colon lying over the aorta. Panel **B—**Proximal colon going down to the pelvis but still on the left of the D-J flexure and of the SB. Panel **C**—Proximal colon going down to the pelvis on the left of the D-J flexure and the SB is only on the right side, no internal hernia. Panel **D—**Middle colic pedicle and its right branch visible going down at the medial side of the colon and aorta. No SB loops are left underneath the colon

**Fig. 5:**
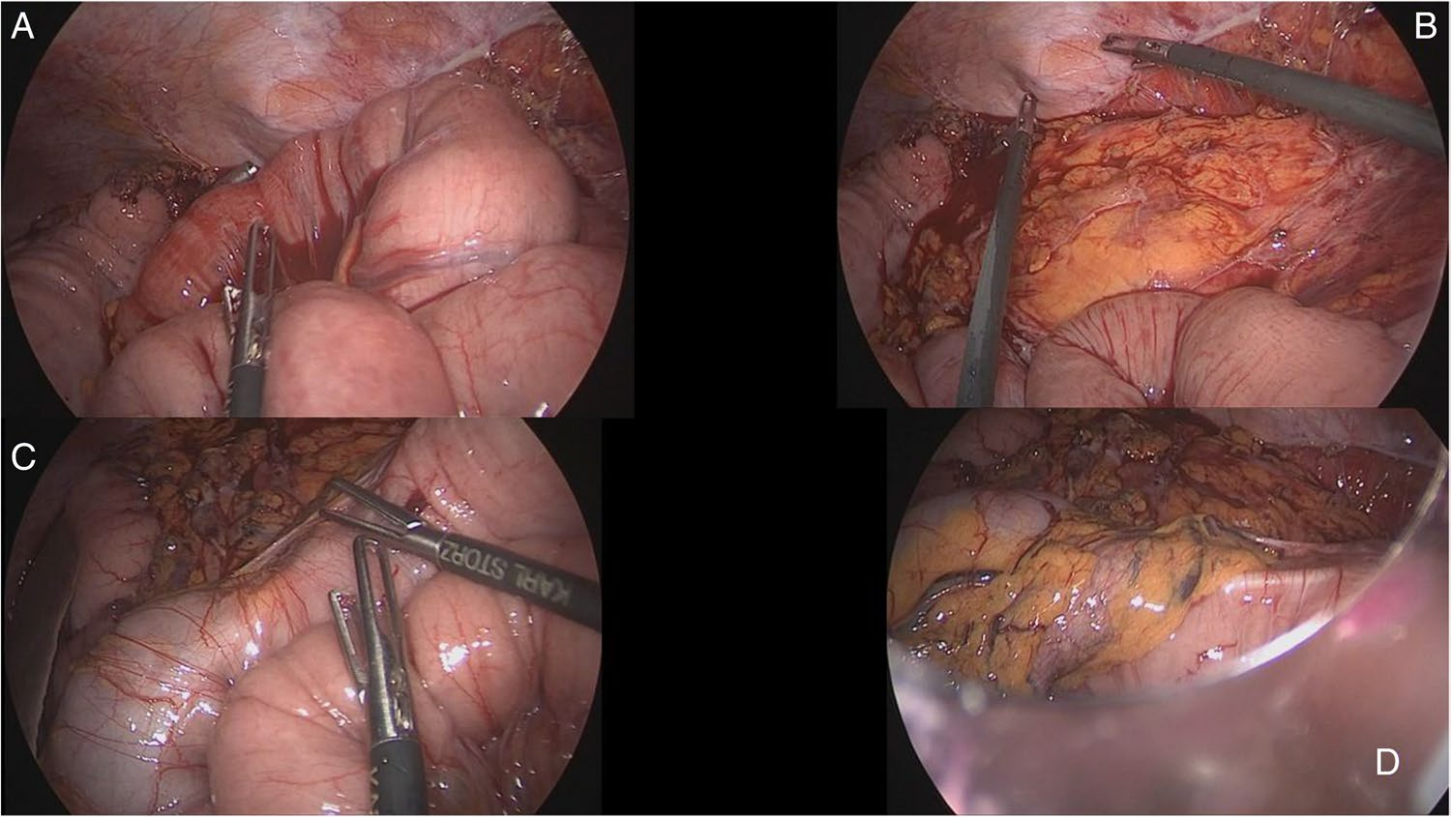
Panel **A**—The SB loops are being pulled towards the right quadrants. Panel **B**—Left quadrants are then free and left without any small bowel. Panel **C**—The proximal colon is going down and care is taken to keep the SB on the right side. Panel **D**—Everything of the small bowel is on the right side. No internal hernias are left behind

This video vignette presents the case of a fit 67-year-old female (BMI 21.2, ASA score 2, hypertension without any other significant comorbidity) presenting with adenocarcinoma of the proximal descending colon. The cancer was impassable by the endoscope and was extending cranially by 7–8 cm on CT scan up to involving the anatomical zone of SF. She had multiple enlarged lymph nodes (LNs) on CT visible along the inferior mesenteric axis and radiological features of visceral serosa involvement (cT4aN + M0). Given her relatively young age, fit conditions, and preoperative features of a locally advanced cancer, she was scheduled for a laparoscopic left extended hemicolectomy (LELC). At laparoscopy, multiple enlarged lymph nodes could be visualized along the IMA and IMV axes. CME with CVL (central vascular ligation) was performed with IMV taken at the level of the inferior border of the pancreas/D-J flexure and IMA taken flush to the aorta. Extended left hemi was completed with full dissection of the transverse mesocolon, identification and dissection of the middle colic vessels, and preservation of the main trunk and of the right branch of the middle colic artery. A full derotation of the right colon was achieved on the embryological planes on a craniocaudal manner from the proximal transverse colon (TC), hepatic flexure, ascending colon, and caecum. Combination of a full release of the fusion fascia of Toldt’s (right retrocolic fascia) and, in the upper medial continuity, release of Fredet’s fascia (fascia preduodenopancreatica) (Fig. 1) allowed for a complete laparoscopic derotation of the remaining right and proximal transverse colon. Then, the specimen (Fig. 2) was delivered through a 5-cm mini-Pfannenstiel incision (muscle-splitting), resection was completed including the left pedicle of middle colics and preserving their right branches, a full mobilization of the right colon and hep flexure was noted (Fig. 3), and 29ch anvil was inserted and secured in the proximal transverse colon stump. A primary intracorporeal transverse-rectal anastomosis was performed. The small bowel mesentery was gently pulled towards the paramedian and right abdominal quadrants, making sure the proximal colonic conduit was lying just lateral of the D-J flexure (which was mobilized itself and appropriately medialized at the time of IMV taking down), avoiding internal hernias and preventing stricture/compression/ischemia of the SB, simply because the colon is not overlying the small bowel loops but it is going down to the pelvis following a longitudinal arcuate pathway. The colon will therefore follow its pathway to the pelvic brim, remaining on the left side of the D-J flexure and of the small bowel loops and overlying the aorta (Fig. 4). The left quadrant of the abdominal cavity remains empty and free of bowel loops without risk of internal hernia (Fig. 5). A small lesion on the liver surface of segment 4 was detected intraoperatively and resected with a laparoscopic wedge resection (histology: benign solitary necrotic nodule, with no evidence of malignancy). Surgery lasted for 348 min including the small liver wedge resection. The patient had an uneventful recovery. Postoperative histology showed pT3 N0 (0/38 LNs), Mx, L0, V0, pn0, and R0. Patient underwent adjuvant chemo and is currently alive with NED.

### Discussion of the embryology and technical steps

The transverse mesocolon is mobile and not rare, especially in thin patients, which can be redundant if appropriately mobilized. Knowledge of the embryology is of paramount importance: the posterior and superior part of the redundant gastrosplenic ligament fuses to the anterior leaf of the dorsal mesentery of the transverse colon to form the transverse mesocolon. The middle colic vessels mark the location of this structure.

The mesenteric component of each flexure is best described in terms of radial and longitudinal axes. The radial axis of the hepatic flexure extends radially from the middle colic vascular pedicle to the intestinal margin of the mesentery. As it does so, the mesentery changes from attached (to the posterior abdominal wall) to non-attached and thus mobile. The longitudinal axis extends longitudinally from the right mesocolon to the transverse mesocolon. At the right mesocolic pole of the longitudinal axis, the mesentery is fully attached across its breadth. At the transverse mesocolic pole of the longitudinal axis, the mesentery is attached centrally but mobile at the intestinal margin. Thus, the mesenteric component of the hepatic flexure undergoes considerable conformational changes. The transverse mesocolon elongates dramatically at the intestinal margin. In this region, and due to elongation, it folds back on to itself and adopts a conformation considerably variable [1].

The transverse mesocolon and colon overlie the small intestinal mesentery, and the greater omentum overlies the upper surface of the transverse mesocolon. Extensive adhesions occur between the under surface of the greater omentum and the upper surface of the transverse mesocolon. As a result, the lesser sac is frequently obliterated where the transverse mesocolon and greater omentum are attached. This arrangement has surgical implications and this is the theoretical basis for our modified technique of allowing the TC to reach the pelvis without the need of sacrificing the middle colic vessels.

As occurs in the right and left mesocolon, mesenteric fat is increased around the middle colic artery (the middle colic adipo-vascular pedicle). On either side of this pedicle, the mesentery thins to the point of being translucent in individuals whose body mass index is low (i.e., the avascular interpedicular regions) [1].

**Fig. 6:**
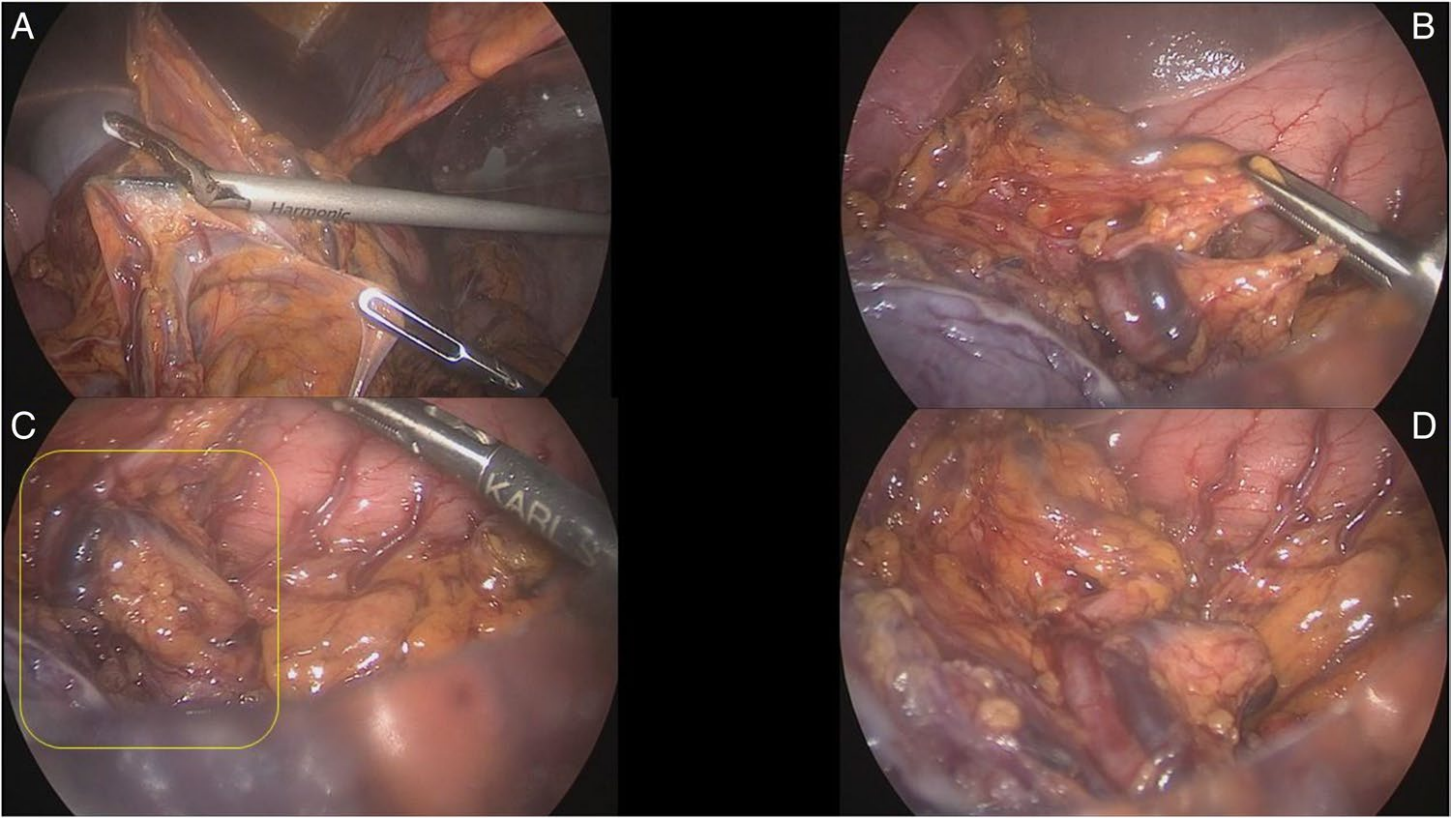
Panel **A**—Dissection of the root of transverse colon mesentery with complete skeletonization and mobilization of the middle colic vessels pedicle aiming to get more length and reach without sacrificing the middle colic vessels. Panel **B**—Middle colic vessels fully mobilized and preserved seen from above (lesser sac). Panel **C**—Detail of the origin of the stump of Middle colic vessels fully mobilized and preserved. Panel **D**—Here It can be appreciated how much of reach has been obtained thanks to the mobilization of the root of TC mesentery and the tortuous long segment of middle colic vessels

A crucial step of this modified Deloyers, aiming to easily achieve a full mobilization of the entire length of the transverse mesentery without the need of sacrificing the middle colic vessels, is to procced in a stepwise manner with the following manouvres: performing the dissection of the colo-epiploic attachment close to the upper border of the TC; entering the lesser sac and then gaining adequate extra length of the transverse mesentery for obtaining enough reach up to the pelvic brim, by combined mobilization of the right colon along the Fredet’s fascia and mobilization of the TC by dissecting the root of its mesentery; opening the adhesions between the superior surface of the mesocolon and the inferior border of the pancreas, up to the origin of the middle colic vessels from the SM vesselsA crucial step of this modified Deloyers, aiming to easily achieve a full mobilization of the entire length of the transverse mesentery without the need of sacrificing the middle colic vessels, is to procced in a stepwise manner with the following manouvres: performing the dissection of the colo-epiploic attachment close to the upper border of the TC; entering the lesser sac and then gaining adequate extra length of the transverse mesentery for obtaining enough reach up to the pelvic brim, by combined mobilization of the right colon along the Fredet’s fascia and mobilization of the TC by dissecting the root of its mesentery; opening the adhesions between the superior surface of the mesocolon and the inferior border of the pancreas, up to the origin of the middle colic vessels from the SM vessels (Fig. 6).

By avoiding the creation of a defect in the SB mesentery and delivering the right colon through to meet the rectum, one may raise the criticism that the colon is simply fully mobilized, but brought down to the rectal stump by pulling it over the small bowel, thus exposing to the risk of creating an internal hernia which may cause future problems. However, with this modified Deloyers which avoids the creation of a retro-ileal window, the proximal colon is going down to the pelvic brim, by staying over the aortic line and lying just on the left of D-J flexure, while keeping the small bowel loops medially on the right and median abdominal quadrants. Early adhesions between the proximal colonic conduit and the retroperitoneal surface will prevent any internal hernia of small bowel.

**Fig. 7:**
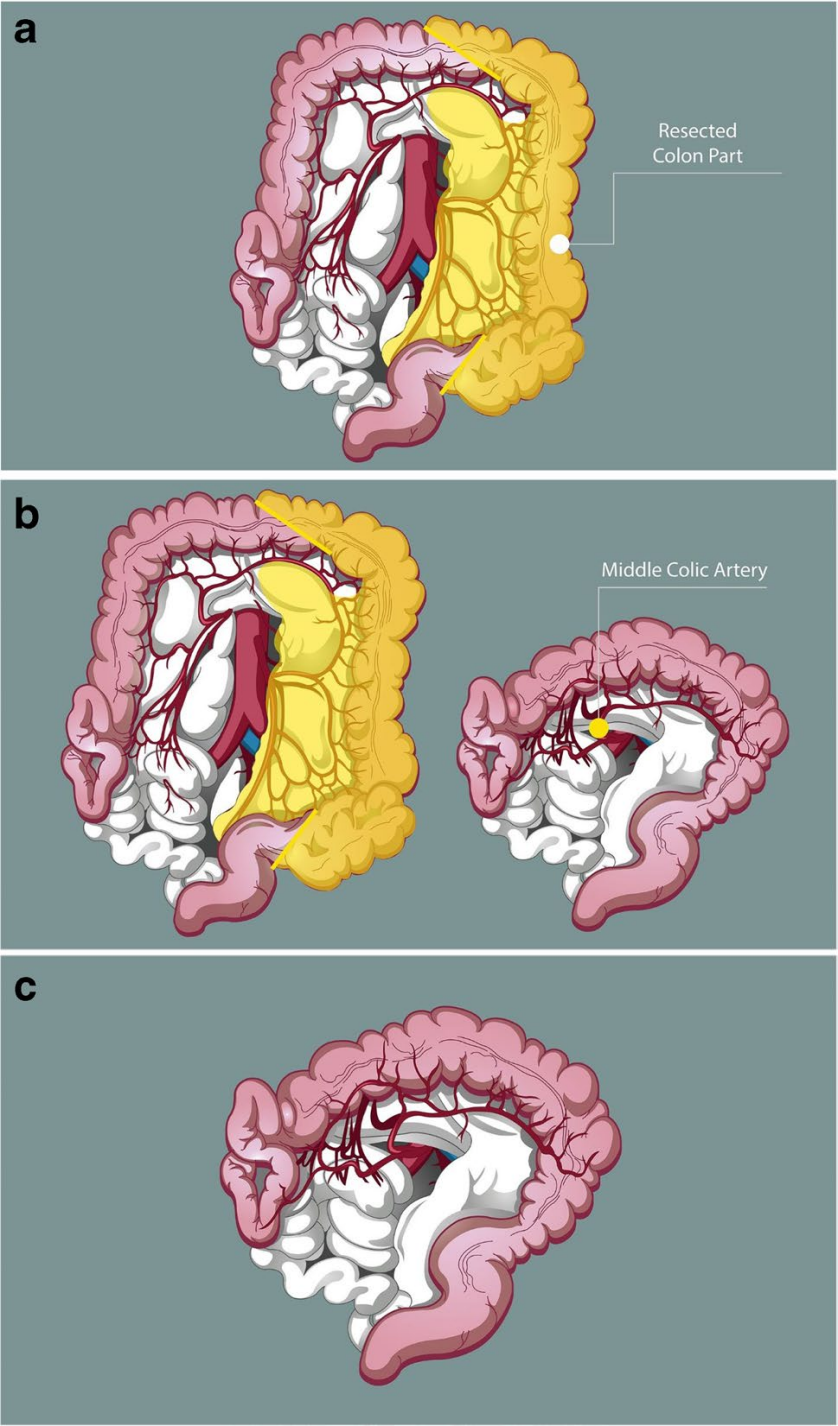
LELC with a primary TC-rectal anastomosis achieved with a partial modified Deloyers by fully mobilizing the transverse colon, with a “middle colic vessel sparing” technique. **a** Laparoscopic extended left colectomy with CME and CVL. **b** Primary anastomosis is achieved by fully mobilizing the transverse colon, with a “middle colic vessel sparing” technique. **c** The fully mobilized mesocolon allows for a tension-free anastomosis while maintaining better blood supply to the mobilized stump and also by eliminating the need for a mesenteric window and the transposition of the caecum; the modified technique allows for the small bowel to rest over the colon and above the anastomosis and by keeping the mobilized transverse colon on the left of the D-J flexure and over the aortic line

While laparoscopic extended right colectomy is a well-established procedure, LELC is rarely used (mainly for distal transverse or proximal descending colon carcinomas extending to the area of the splenic flexure) [2, 3]. Recent nationwide retrospective studies have shown that segmental colonic resection may be a safe and effective alternative in terms of both postoperative and oncological outcomes and was performed more frequently using a minimally invasive approach than extended procedures [4]. However, in selected cases and especially in locally advanced cancers (e.g., cT4), several authors do prefer extended right or extended left colectomies [5]. The most recent meta-analysis comparing all surgical techniques has shown similar oncological outcomes following a segmental, extended left, or subtotal colectomy; however, extended resections achieved a higher number of lymph nodes harvested, higher rate of primary anastomosis, and a trend towards lower rates of anastomotic leak that any of the surgical procedures (segmental, extended or subtotal colectomies) for the curative resection of splenic flexure tumors provide similar survival; however, extended resections are associated with higher number of lymph nodes retrieved (despite no differences among techniques were seen in terms of proportion of patients achieving the minimum threshold of 12 LNs), with a higher rate of primary anastomosis and a non-significant trend for lower anastomotic dehiscence when compared with more restrict resections [6]. The authors conclude that an individualized surgical plan considering both short- and long-term outcomes is necessary to select the appropriate operation. For young patients with locally advanced tumors affecting long colonic segments, extended colectomies may be a better option for achieving a good quality specimen, especially in high-volume centers with more experienced laparoscopic colorectal surgeons. As shown in our patient’s CT scan, the tumor was extending for a long segment and a more aggressive and extended approach may be therefore justified and used in tertiary centers with advanced laparoscopic colorectal skills and expertise and in patients relatively young with preoperative or intraoperative features/suspicion of locally advanced cancers, especially if long colonic segments, extended colectomies maybe preferable and achieve better oncological radicality, both in terms of nodal clearance (including the IMA axis as in this case) and in terms of margins of resection (as you can see from CT in this patient, the tumor was extending for a long segment). LELC (Fig. 7a) carries several technical challenges which are demonstrated in this video vignette. Firstly, the steps needed to mobilize the left colon and procure a safe approach to the splenic flexure are described, especially when a tumor is closely related to it. This is achieved by mobilization and resection of the descending colon, while maintaining a complete mesocolic excision [7] to the level of the duodenojejunal ligament for the inferior mesenteric vein and flush to the aorta for the inferior mesenteric artery. Subsequently, we describe the operative steps required to enable a primary anastomosis by fully mobilizing the transverse colon, with a “middle colic vessel sparing” technique (Fig. 7) and release as much of the mesocolic attachments at the splenic flexure area.

In conclusion, we present the rare instance when a laparoscopic derotation of the ascending colon is required to provide a tension-free anastomosis. The resection is completed by delivering of the fully derotated ascending colon and hepatic flexure through a suprapubic mini-Pfannenstiel incision. The primary colorectal anastomosis is subsequently fashioned in a tension-free way and provides for a quick postoperative recovery of the patient. This modified and partial Deloyers procedure preserves the middle colic since the fully mobilized mesocolon allows for a tension-free anastomosis while maintaining better blood supply to the mobilized stump (Fig. 7b, c). Also, by eliminating the need for a mesenteric window and the transposition of the caecum, it allows the small bowel to rest over the colon and above the anastomosis and by keeping the mobilized transverse colon on the left of the D-J flexure, and over the aortic pathway, the possibility of an internal herniation of the small bowel into the mesentery is virtually none because the proximal colon conduit is going down to the pelvis, and gets immediately adherent to the retroperitoneal surface, where the left CME has been performed (Fig. 7c).

Laparoscopic derotation of the right colon is a feasible technique in experienced hands to provide primary anastomosis after LELC with improved functional outcome. Nevertheless, it is important to consider anatomical aspects of the left hemicolectomy along with oncological considerations, to provide both a safe oncological resection along with good postoperative bowel function.

## Supplementary Information

Below is the link to the electronic supplementary material.

Supplementary file1 (PPTX 145079 KB)

Supplementary file2 (PPTX 145079 KB)

Supplementary file3 (DOCX 20 KB)

Supplementary file4 (DOCX 50.5 KB)
