# Supplementary material for: Long splenic flexure carcinoma requiring laparoscopic extended left hemicolectomy with CME and transverse-rectal anastomosis: technique for a modified partial Deloyers in 5 steps to achieve enough reach and preserving middle colic vessels
Source: Langenbecks Arch Surg. 2021 Jul 16;407(1):421–8. doi: 10.1007/s00423-021-02240-7 (PMC8847254; doi:10.1007/s00423-021-02240-7)
Supplement: Supplementary file 3 — Supplementary file3 (DOCX 20 KB) [file 423_2021_2240_MOESM3_ESM.docx]

**Long splenic flexure carcinoma requiring Laparoscopic extended left hemicolectomy with CME and transverse-rectal anastomosis: technique for a modified partial Deloyers in 5 steps to achieve enough reach and preserving middle colic vessels**

VIDEO **PRIVATE LINK** FOR THE EDITOR AND PEER REVIEWERS

**REVISED VERSION OF THE VIDEO ACCORDING TO REVIEWERS COMMENTS: MAY 2021**

<https://youtu.be/IUWJfIyxUZo>
