# Supplementary material for: Long splenic flexure carcinoma requiring laparoscopic extended left hemicolectomy with CME and transverse-rectal anastomosis: technique for a modified partial Deloyers in 5 steps to achieve enough reach and preserving middle colic vessels
Source: Langenbecks Arch Surg. 2021 Jul 16;407(1):421–8. doi: 10.1007/s00423-021-02240-7 (PMC8847254; doi:10.1007/s00423-021-02240-7)
Supplement: Supplementary file 4 — Supplementary file4 (DOCX 50.5 KB) [file 423_2021_2240_MOESM4_ESM.docx]

**supplementary material 4: [https://youtu.be/IUWJfIyxUZo](https://urldefense.proofpoint.com/v2/url?u=https-3A__youtu.be_IUWJfIyxUZo&d=DwQFaQ&c=vh6FgFnduejNhPPD0fl_yRaSfZy8CWbWnIf4XJhSqx8&r=F8jRe85hu55p0hrOAPRTlXqBWmdi_A5USzQxvJXlzL2tJvyEWDgfrhKJOIzcdlym&m=aMnzT3TklU7zB7xfc2ziPq1mpp2bngj9T_BVl0Oi4Xo&s=aSqnQvud1Ste7x6LiHyLKoyC0B7MObWhbSlZ8LcSitE&e=)**

educational surgical technique How-I-Do-It associated with the following article

https://doi.org/10.1007/s00423-021-02240-7 or as a PDF here https://link.springer.com/content/pdf/10.1007/s00423-021-02240-7.pdf

Title

Long splenic flexure carcinoma requiring laparoscopic extended left hemicolectomy with CME and transverse-rectal anastomosis: technique for a modified partial Deloyers in 5 steps to achieve enough reach and preserving middle colic vessels

Journal Langenbeck's Archives of Surgery DOI 10.1007/s00423-021-02240-7 Publication Date 2021-07-16
